# Development and pilot evaluation of a mobile app on parent-child exercises to improve physical activity and psychosocial outcomes of Hong Kong Chinese children

**DOI:** 10.1186/s12889-020-09655-9

**Published:** 2020-10-14

**Authors:** Rosa Sze Man Wong, Esther Yee Tak Yu, Thomson Wai-Lung Wong, Colman Siu Cheung Fung, Cynthia Sin Yi Choi, Calvin Ka Lun Or, Kiki Sze Nga Liu, Carlos King Ho Wong, Patrick Ip, Cindy Lo Kuen Lam

**Affiliations:** 1 Department of Family Medicine and Primary Care, The University of Hong Kong, 3/F, Ap Lei Chau Clinic, 161 Ap Lei Chau Main Street, Ap Lei Chau, Hong Kong, Hong Kong; 2 Department of Paediatrics and Adolescent Medicine, The University of Hong Kong, Hong Kong, Hong Kong; 3 School of Public Health, The University of Hong Kong, Hong Kong, Hong Kong; 4 Department of Industrial and Manufacturing Systems Engineering, The University of Hong Kong, Hong Kong, Hong Kong

**Keywords:** Physical activity, Smartphone application, Health-related quality of life, Exercise, Behavior, Parent-child interaction, Mhealth interventions

## Abstract

**Background:**

Parent-child exercises involve children and parents to do workout together and have positive effects on physical and mental health. We developed a mobile app on parent-child exercises called Family Move, which combines coaching videos with game features such as points and level system to enhance the health and wellbeing of both children and parents through parent-child exercises. This pilot pre-post study investigated whether the Family Move app-based intervention had a positive effect on children’s health-related quality of life (HRQOL), psychosocial wellbeing, and physical activity (PA) level.

**Methods:**

We recruited 67 parent-child pairs. During the 8-week intervention, these pairs were invited to perform parent-child exercises using the Family Move app. Points were automatically added to the user account after viewing a coaching video. In-game ranking was available to enhance user engagement. Parent proxy-report questionnaires on children’s HRQOL, psychosocial wellbeing, and PA were administered at baseline and 1- and 6-month follow-up. Paired samples t-tests were conducted to evaluate post-intervention changes in child outcomes (HRQOL, psychosocial wellbeing, and PA). Multiple linear regressions were used to examine these changes as a function of in-game ranking.

**Results:**

52 (78%) viewed at least one coaching video in the Family Move app. Children’s PA level significantly increased at 1-month (d = 0.32, *p* = 0.030) and 6-month (d = 0.30, *p* = 0.042) follow-up, whereas their psychosocial problems declined at 6-month follow-up (d = 0.35, *p* = 0.005). Higher in-game ranking was significantly associated with fewer psychosocial problems at 1-month follow-up (β = − 0.15, *p* = 0.030).

**Conclusions:**

Our findings suggest that the Family Move app could be a possible intervention to increase children’s PA level and psychosocial wellbeing through parent-child exercise.

**Trial registration:**

ClinicalTrials.gov number, NCT03279354, registered September 11, 2017 (Prospectively registered).

## Background

Physical activity (PA) is important for good health and quality of life. Regular PA is associated with general health, vitality, physical functioning, and mental health [1]. Research has demonstrated the effectiveness of PA interventions to improve health and wellbeing [2–5]. However, some individuals are more reluctant to initiate an active lifestyle for reasons such as low exercise motivation, insufficient exercise knowledge, lack of time, and unaffordable cost of programs/facilities [6]. For example, the Active Healthy Kids 2018 Hong Kong Report Card showed that despite a generally favorable community environment, children and youth in Hong Kong, especially those in lower socioeconomic families [7], remained low in PA and physical fitness levels and high in sedentary behaviors [8]. The 2013 territory-wide study on the physical fitness of Hong Kong people revealed that a large proportion of children (48.2%) and adolescents (57.7%) did not participate in moderate to vigorous physical activities (MVPA) at least 3 days a week with accumulation of 30 min or above per day [9]. Indeed, physical inactivity has been increasing in many countries posing a serious threat to global public health [10]. While international PA guidelines recommend at least 60 min of MVPA daily for children and adolescents, it should be noted that doing amounts below the recommended levels can still be beneficial especially for inactive children who are advised to start with small amounts to build up their interest in PA over time [11]. Evidence also shows that physical inactivity can be passed from generation to generation. Inactive parents are less likely to encourage their children to engage in PA and spend less time in activities with children [12]. Lack of parental facilitation may increase children’s risk of physical inactivity [13, 14]. On the other hand, getting active with parents can boost children’s PA interest and behavior [15]. Hence, parent-child exercises have the potential to engage children in regular PA habit.

Parent-child exercise is an extended form of traditional exercise that requires children and parents to do workout so both parents and children can benefit from an active lifestyle together [16]. In addition, the fun and joyful time during parent-child exercises not only brings family together but also motivates inactive children to get active. However, most of the existing PA programs are conducted in schools and target at students without involving their family members. In particular, parents facing competing demands between work and family would have greater difficulty attending school- or center-based parent-child exercise programs [17]. Hence, there is a need to develop a set of simple parent-child exercises for inactive parents and children to get active in their own setting even without training equipment.

In recent years, many health and fitness apps have been developed to improve lifestyle behavior [18], but most of the available exercise apps are tailored for Western populations and in English, which reduces their applicability to Chinese families. The few PA promotion apps that are available in Chinese language mainly target at individual adult users without considering whether the exercise moves are appropriate for children, and none of them provides instructions on parent-child exercises, which make it difficult to use in a family intervention. There is evidence that supports the feasibility of a 6–8-week home-based exercise intervention in improving the health-related quality of life (HRQOL) of patients with pediatric cancer [19]. However, the few studies investigating the effectiveness of PA app-based interventions for children and adolescents have reported inconsistent findings [20–23]. Hence, the aim of this study was to conduct a pilot study to examine the effect of the Family Move app-based intervention on children’s health-related quality of life (HRQOL), psychosocial wellbeing, and PA levels. We also examined these changes as a function of the in-game ranking.

## Methods

### Theoretical basis

The Family Move app incorporated multiple health behavior theory constructs and strategies. The major source of theoretical guidance for the app comes from the social cognitive theory (SCT) which posits that people learn by watching others through observation, imitation, and modeling [24]. It also emphasizes the reciprocal relationship of personal (e.g. financial and weight problems) and environmental factors (e.g. limited availability and accessibility of PA resources) with behaviors (e.g. PA behavior). Evidence suggests that observational learning, self-regulation, psychological states, and environmental characteristics are determinants of behaviors [25]. A key psychological determinant is self-efficacy which refers to “beliefs in one’s capabilities to mobilize the motivation, cognitive resources, and courses of action needed to meet given situational demands” [26]. Another psychological determinant is outcome expectations defined as “one’s judgement of the likely consequences that will occur as a result of performing, or not performing, a particular behavior” [27]. External factors such as incentive motivation and use of special app functions to engage participants can also influence behaviors [25].

**Fig. 1:**
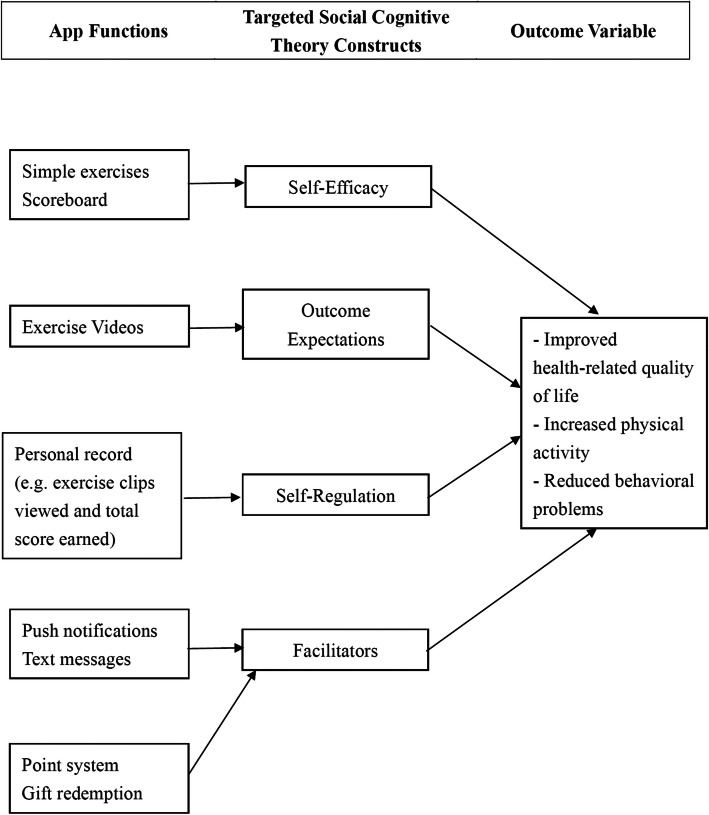
Theoretical framework of the Family Move app

The SCT has been used to guide the development of website intervention for promotion of PA in young adults [28]. This study adopted a similar conceptual framework. Figure 1 illustrates the model describing how the Family Move app functions affect the target outcomes in children through their effects on different SCT constructs:

- *Self-efficacy:* Simple exercise moves and a scoreboard documenting user effort and participation in the form of accumulated points were included to increase user motivation and confidence in performing the exercise moves.
- *Outcome expectations*: Joyful interactions between two people (demonstrated in the exercise video) and specific fitness benefits (in the form of an audio instruction) were used to enhance positive outcome expectations in users.
- *Self-regulation*: A Personal Record page was included to allow users to monitor and regulate their own performance with reference to peer performance.
- *Facilitators*: Gaming elements such as point system and reward system are incorporated into the Family Move app to increase user engagement. Push notifications and text messages about exercise benefits are also used to engage users to use the app

In addition, we also used other motivational elements and principles such as developmentally appropriate exercises, gamification, professional endorsement, and data privacy to attract people to use the app.

### Development process

The development and testing phases of the Family Move app were conducted from August 2016 to May 2017 by a collaborative team of physiotherapists, clinicians, fitness trainers, health promotion information technology experts, and graphic designers. The Family Move app incorporates coaching videos with gaming elements such as points, level system, and scoreboard for tracking progress among users [29]. Given that our target participants may have varying degrees of physical abilities and fitness levels, the expert panel decided to develop a staged intervention with short bursts of exercises (i.e. 30 s per exercise move) at three difficulty levels (basic, intermediate, and advanced). During August–September 2016, a list of simple stretching and aerobic exercises stratified by child age (6–10 years and 11 years or above) were selected and modified to partner exercise format by PA specialists. In the subsequent pilot course, 14 parent-child pairs tried to perform the shortlisted exercises under the instructions of a certified fitness trainer. During the course, parents and children demonstrated high interest in basic aerobic exercises and enjoyed the parent-child interaction time. However, parents found stretching exercises difficult to perform because of the physical demands for muscle strength and flexibility. They also expressed concerns over extension exercises because of limited living space. Based on these feedbacks, the exercise moves were further adjusted to meet these needs of parents and children before filming the moves.

The exercise video production process began in December 2016. We recorded a total of 18 exercise moves which were demonstrated by a certified fitness trainer and a child with good athletic skills. During post-production, the coaching videos were further edited with the addition of background music, timer, color correction, and subtitles highlighting the health benefits of the exercise move. Each in-game level would have a warm-up exercise clip, four independent exercise move clips, and a three-minute challenge clip that allows for interval training [30]. The details of the coaching videos are provided in Additional File 1.

After viewing a coaching video in the app, users would automatically receive points, with more points being awarded to more difficult moves. Furthermore, to create a competition context, users would be ranked by the total number of points earned through viewing the coaching videos. The top five frequent users’ scores would be displayed anonymously in the in-app scoreboard. In addition to gaming elements, push notifications and text messages would also be used to enhance user motivation for participation. Based on the level of usage recorded in the Central Management System (CMS), psychoeducational text messages can be tailored and sent to different groups of users (i.e., logged-in and active users, logged-in yet inactive users, and never logged-in users) on a regular basis. For example, messages for inactive users would focus on the fun parts of the app and associated benefits (e.g. *Forward and backward lunge can serve as balance training to strengthen the muscles of thigh, leg and hip; the demo clip is now available in FAMILY MOVE!*), whereas messages for active users would focus on encouragement by acknowledging the user’s good progress (e.g. *Good job, you are making excellent progress!*). Screenshots and details of the functions of the Family Move app are provided in Additional File 2.

Basic software requirements were also confirmed during this development period. For example, it was confirmed that the app would operate in devices running iOS 9.0–10.0 and Android 4.4.2–6.0 and require username and password for access. This user registration function is needed to record a user’s login time and usage. These records can be retrieved from the password-secured CMS where administrators can configure, manage, and monitor user accounts.

### Study design

A pilot study using the pre-post assessment method was designed to examine changes in children’s HRQOL, psychosocial wellbeing, and PA levels after the Family Move app-based intervention. The intervention lasted 8 weeks with three assessment time points (baseline, 1-month and 6-month follow-up).

### Ethics

Ethics approval was granted by the Institutional Review Board of the University of Hong Kong/Hospital Authority Hong Kong West Cluster (UW 17–179).

### Study population

Families with at least one child aged 6 to 15 years were targeted, as children in this age period should develop the physical competence to perform exercise moves and still enjoy spending time with parents. Eligible families must be ethnically Chinese and live in Hong Kong. In addition, the parents must have a smartphone that can download and install apps and provide consent for themselves and their children to participate. To maximize the social diversity of our sample and ensure adequate sample size, we recruited participants from local schools and community programme through convenience sampling. Specifically, we approached 19 local schools, three of which expressed interest to help with recruitment by distributing relevant study information to their students; then our research assistant briefed the interested students about the intervention through phone calls or text messages. Meanwhile, we also advertised our study in the newsletter of the community low-income family support program which was conducted by our team in partnership with a philanthropy foundation, the Kerry Group Kuok Foundation [31]. Given that the effect size of d = 0.3 is usually considered as the minimal clinically important difference in HRQOL [32], the power analysis showed that a minimum of 34 parent-child pairs would be sufficient to detect such effect size with 80% power at the 5% significance level.

### Data collection

Parents of eligible children were given information about the objectives and procedure of the study. Interested parents subsequently provided written consent separately for themselves and for their children to participate in this study. Instructions with the link to download and install the app onto their smartphone and the family-specific login information were sent to parents by text message. Parents were asked to complete a set of parent-proxy report questionnaires on their children’s HRQOL, psychosocial wellbeing, and PA level at baseline and 1- and 6-month follow-up. They were reminded to mail it to the research team office within 2 weeks after distribution.

### Intervention

Each participating family had an account in the Family Move app. The login information was sent to the parent by text message. During the 8-week intervention period, participating families received parent-child exercise training through the Family Move app by stages (i.e. basic training in the first 2 weeks, intermediate training in the third and fourth week, advanced training in the fifth and sixth week, and no training restriction in the final 2 weeks). Each coaching video included Cantonese audio instructions that guided parent participants to perform the exercise move with their children while watching and encouraged them to incorporate the move into their daily routine. Prior to the intervention, all participants were informed that families who earned at least 5000 points over the intervention period would be given a gift worth HK$50 (US$ 6.4) for their active participation in the intervention.

### Measures

#### Health-related quality of life

The Chinese Child Health Questionnaire – Parent Form – 50 (CHQ-PF50) was used to assess HRQOL. The tool includes twelve domain scales (General Health, Physical Functioning, Role/Social Limitations – Emotional/Behavioral, Role/Social Limitations – Physical, Bodily Pain/Discomfort, Behavior, Mental Health, Self-Esteem, Parental Impact – Emotion, Parental Impact – Time, Family Activities, and Family Cohesion) and two summary scores (Physical Summary Score and Psychosocial Summary Score) [33]. Higher scores indicate better HRQOL.

#### Psychosocial wellbeing

The Chinese Strength and Difficulties Questionnaire (SDQ) was used to assess psychosocial wellbeing. The tool includes five subscales of emotional symptoms, conduct problems, hyperactive/inattentive problems, prosocial behaviors, and peer relationship problems [34]. Higher scores indicate more problem behavior for all except the prosocial behavior score. The scores of the four problem behavior subscales can be summated to a total difficulties score.

#### Physical activity level

The International Physical Activity Questionnaire – Short Form - Chinese version (IPAQ-SF) was used to assess physical activity level. The tool includes seven questions on the frequency and duration of time spent in physical activity in the past 7 days [35] to calculate total weekly PA level expressed in MET-minutes/week.

#### Family move app usage

The Family Move app usage was indicated by the total number of points earned through viewing the coaching videos in the app over the 8-week intervention period. This usage information can be retrieved from the CMS. Higher points indicate more frequent use of the Family Move app. We categorized participants as users (those parent-child pairs who had viewed at least one video clip during the intervention period) and non-users (those parent-child pairs who had never viewed a coaching video during the intervention period).

### Data analysis

All analyses were carried out using IBM SPSS statistical 24.0 Version. Descriptive statistics were used to describe the characteristics of the study sample. Data were analyzed using the intention-to-treat (ITT). Last observation values were carried forward to impute the missing data. Shapiro-Wilk test revealed normal distribution for all continuous variables at baseline and follow-ups. Paired sample t-tests were used to examine changes in CHQ-PF50 scores, SDQ scores, and total IPAQ-SF scores in MET-minutes/week at 1 month and 6 months after the intervention. The effect size expressed as Cohen’s d was used to standardize the estimates of outcome changes to allow for comparison between studies.

To explore the association between engagement with the Family Move app and outcomes changes post intervention in children, participants were ranked based on the amount of points earned over the 8-week intervention. Higher rank indicates more frequent views of the coaching videos in the app. After the 8-week intervention, the highest rank was 42 which corresponded to 9650 points. Details of the user ranking can be found in Additional File 3. After creating the participation ranking variable, multiple linear regression analyses were performed with participation ranking as the independent variable and post-intervention outcome changes as the dependent variable, adjusting for child gender, age and baseline outcome level and recruitment month. All regression coefficients were standardized, with *p* value less than 0.05 indicating statistical significance.

## Results

### Participant flow and recruitment

**Fig. 2:**
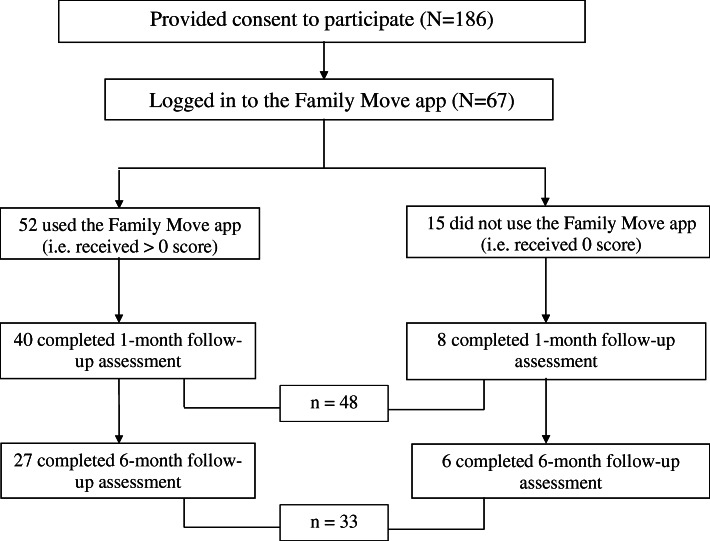
Subject flowchart

Recruitment began from May to December 2017. A total of 186 parent-child pairs consented to participate in this study and completed the baseline survey. The subject flowchart is presented in Fig. 2. This study involved 67 (36%) who had logged in to the app. Among them, 52 (78%) viewed at least one coaching video in the app. Among the users, 40 completed the 1-month survey and 27 completed the 6-month follow-up survey. Among the non-users, 8 completed the 1-month survey and 6 completed the 6-month follow-up survey.

### Sample characteristics

Tables 1 and 2 shows the differences in baseline characteristics between users and non-users. At baseline, participants had a mean age of 10.52 years (SD 2.1), and 13 (19%) were female. The participants had a wide range of monthly household income, where the bottom 25% was below HKD 18,000 (US$ 2308) and the top 25% was above HKD 100,000 (US$ 12,820). Their average month household income was HKD 73,050 (US$ 9131). Children spent 4.5 h per week, on average, engaging in vigorous and moderate activities. There were no statistically significant differences between users and non-users in terms of parental age, gender and education level, family household income, and child gender and age.

**Table 1:** Subject characteristics

	Overall (*n* = 67)	Users (*n* = 52)	Non-Users (*n* = 15)	*p*-value
Parent gender, n(%)				–
Female	67 (100%)	52 (100%)	15 (100%)	
Male	0 (0%)	0 (0%)	0 (0%)	
Parent age, mean(SD)	43.13 (5.22)	43.46 (5.04)	42.00 (5.84)	0.343
Parent education level, n(%)				0.059
Primary education or below	4 (6.00)	3 (5.90)	1 (7.70)	
Secondary education	25 (37.30)	18 (35.30)	7 (53.80)	
Post-secondary education	6 (9.00)	3 (5.90)	3 (23.10)	
Tertiary education	29 (43.30)	27 (52.90)	2 (15.40)	
Missing	3 (4.50)	–	–	
Parent employment, n(%)				0.002
Employed	35 (52.20)	33 (64.70)	2 (15.40)	
Unemployed	29 (43.30)	18 (35.30)	11 (84.60)	
Missing	3 (4.50)	–	–	
Child gender, n(%)				0.146
Female	13 (19.40)	8 (15.38)	5 (33.33)	
Male	54 (80.60)	44 (84.62)	10 (66.67)	
Child age, mean(SD)	10.52(2.10)	10.38(2.20)	11.00(1.69)	0.321
Monthly household income (HKD ‘000), mean(SD)	73.05(88.56)	74.02 (73.81)	69.11(137.80)	0.865

**Table 2:** Child baseline health-related characteristics

	Overall (*n* = 67)	Users (*n* = 52)	Non-Users (*n* = 15)	
	mean (SD)	mean (SD)	mean (SD)	*p*-value
**IPAQ-SF**				
Total physical activity level (MET-minutes/week)	2732.44 (2362.34)	2697.95 (2474.19)	2889.56 (1881.36)	0.828
Time spent in vigorous and moderate activities (minutes/week)	282.86 (293.52)	285.78 (314.60)	270.91 (195.37)	0.882
**CHQ-PF50**				
Physical functioning	96.02(10.85)	96.9(8.55)	92.96(16.6)	0.218
Role functioning - emotional/behavior	87.73(21.11)	89.1(19.12)	82.96(27.17)	0.325
Role functioning - physical	95.27(13.55)	94.87(14.59)	96.67(9.34)	0.655
Bodily pain	87.16(17.39)	87.50(16.19)	86.00(21.65)	0.771
General behavior	76.21(16.63)	77.28(16.56)	72.50(16.88)	0.331
Mental health	81.04(14.37)	82.12(11.13)	77.33(22.43)	0.437
Self-esteem	70.09(13.21)	70.75(11.94)	67.78(17.21)	0.447
General health perceptions	68.16(18.01)	69.09(17.47)	64.94(20.1)	0.437
Parental impact: emotional	78.61(21.38)	79.65(20.17)	75.00(25.59)	0.463
Parental impact: time	80.43(23.62)	81.2(22.28)	77.78(28.48)	0.625
Family activities	81.03(23.05)	81.65(22.22)	78.89(26.47)	0.686
Family cohesion	67.73(23.57)	68.82(23.14)	64.00(25.44)	0.490
Physical Summary Score	53.19(7.69)	53.38(7.25)	52.53(9.32)	0.711
Psychosocial Summary Score	49.21(9.28)	49.92(8.07)	46.77(12.66)	0.375
**SDQ**				
Emotional symptoms	1.88(1.99)	1.75(1.72)	2.33(2.74)	0.450
Conduct problems	1.73(1.46)	1.62(1.33)	2.13(1.85)	0.230
Hyperactivity/inattention	3.39(2.30)	3.35(2.14)	3.53(2.88)	0.784
Peer relationship problems	2.39(2.01)	2.23(1.85)	2.93(2.46)	0.235
Prosocial behavior	7.16(1.96)	7.19(2.02)	7.07(1.79)	0.829
Total difficulties	9.39(5.25)	8.94(4.45)	10.93(7.4)	0.198

*CHQ-PF50* Child Health Questionnaire-Parent Form-50; *IPAQ-SF* International Physical Activity Questionnaire – Short Form; *SDQ* Strengths and Difficulties Questionnaire

### Outcomes at 1- and 6-month follow-up

The month of recruitment had no association with outcome changes at 1- or 6-month follow-up. Table 3 displays the IPAQ-SF score, CHQ-PF50 scale and summary scores, and SDQ subscale and total difficulties scores among the 67 pairs at baseline and 1-month follow-up. There was a statistically significant increase in total PA level (d = 0.32, *p* = 0.030) and time spent in vigorous and moderate activities (d = 0.28, *p* = 0.040) at 1-month follow up. Table 4 displays the scores at 6-month follow-up. There was a statistically significant increase in total PA level (d = 0.30, *p* = 0.042) and CHQ-PF50 General Behavior (d = 0.28, *p* = 0.023) and General Health Perceptions scale score (d = 0.34, *p* = 0.007) and a statistically significant decrease in SDQ Total Difficulties score (d = − 1.19, *p* = 0.005), with greater improvement found in SDQ Hyperactivity/Inattention (d = − 0.52, *p* = 0.002) and Prosocial Behavior scores (d = 0.63, *p* = 0.003).

**Table 3:** Changes in outcome measure scores at 1-month follow-up (*n* = 67)

	Baseline Mean (SD)	1 month Mean (SD)	d	*p*-value
**IPAQ-SF**				
Total physical activity level (MET-minutes/week)	2732.44(2362.34)	3363.61(2674.95)	0.32	0.030
Time spent in vigorous and moderate activities (hours/week)	282.86(293.52)	360.8(324.49)	0.28	0.040
**CHQ-PF50**				
Physical functioning	96.02(10.85)	96.85(7.32)	0.10	0.405
Role functioning - emotional/behavior	87.73(21.11)	88.06(20.04)	0.02	0.864
Role functioning - physical	95.27(13.55)	95.27(13.86)	0.00	1.000
Bodily pain	87.16(17.39)	87.16(16.22)	0.00	1.000
General behavior	76.21(16.63)	77.36(18)	0.10	0.438
Mental health	81.04(14.37)	79.7(16.85)	0.15	0.236
Self-esteem)	70.09(13.21)	70.15(14.13)	0.01	0.957
General health perceptions	68.16(18.01)	69.98(16.67)	0.14	0.272
Parental impact: emotional	78.61(21.38)	80.6(19.91)	0.14	0.241
Parental impact: time	80.43(23.62)	81.59(23.42)	0.09	0.481
Family activities	81.03(23.05)	83.52(22.56)	0.14	0.248
Family cohesion	67.73(23.57)	69.39(24.28)	0.09	0.469
Physical summary score	53.19(7.69)	53.75(6.65)	0.11	0.385
Psychosocial summary Score	49.21(9.28)	49.38(10.28)	0.03	0.797
**SDQ**				
Emotional symptoms	1.88(1.99)	1.79(1.99)	0.08	0.495
Conduct problems	1.73(1.46)	1.85(1.71)	0.10	0.398
Hyperactivity/inattention	3.39(2.30)	3.1(2.21)	0.22	0.079
Peer relationship problems	2.39(2.01)	2.34(1.81)	0.03	0.804
Prosocial behavior	7.16(1.96)	7.48(1.93)	0.22	0.081
Total difficulties	9.39(5.25)	9.09(5.27)	0.11	0.388

*CHQ-PF50* Child Health Questionnaire-Parent Form-50; *IPAQ-SF* International Physical Activity Questionnaire – Short Form; *SDQ* Strengths and Difficulties Questionnaire

**Table 4:** Changes in outcome measure scores at 6-month follow-up (*n* = 67)

	Baseline Mean (SD)	6 months Mean (SD)	d	*p*-value
**IPAQ-SF**				
Total physical activity level (MET-minutes/week)	2732.44(2362.34)	3448.28 (3043.95)	0.30	0.042
Time spent in vigorous and moderate activities (hours/week)	282.86(293.52)	343.21 (343.14)	0.21	0.126
**CHQ-PF50**				
Physical functioning	96.02(10.85)	96.52(11.31)	0.04	0.763
Role functioning - emotional/behavior	87.73(21.11)	88.23(20.82)	0.03	0.827
Role functioning - physical	95.27(13.55)	95.02(15.36)	0.02	0.888
Bodily pain	87.16(17.39)	88.36(15.14)	0.07	0.543
General behavior	76.21(16.63)	80.21(17.06)	0.28	0.023
Mental health	81.04(14.37)	80.67(16.44)	0.03	0.776
Self-esteem	70.09(13.21)	71.27(15.03)	0.13	0.278
General health perceptions	68.16(18.01)	72.45(16.47)	0.34	0.007
Parental impact: emotional	78.61(21.38)	79.98(19.89)	0.09	0.475
Parental impact: time	80.43(23.62)	83.42(23.64)	0.17	0.164
Family activities	81.03(23.05)	85.07(22.07)	0.20	0.102
Family cohesion	67.73(23.57)	69.24(25.01)	0.07	0.579
Physical summary score	53.19(7.69)	53.9(7.39)	0.11	0.379
Psychosocial summary score	49.21(9.28)	50.22(10.3)	0.17	0.181
**SDQ**				
Emotional symptoms	1.88(1.99)	1.58(1.99)	0.27	0.028
Conduct problems	1.73(1.46)	1.45(1.56)	0.21	0.095
Hyperactivity/inattention	3.39(2.30)	2.87(2.12)	0.40	0.002
Peer relationship problems	2.39(2.01)	2.3(1.78)	0.05	0.675
Prosocial behavior	7.16(1.96)	7.79(1.91)	0.37	0.003
Total difficulties	9.39(5.25)	8.19(5.31)	0.35	0.005

CHQ-PF50: Child Health Questionnaire-Parent Form-50; IPAQ-SF: International Physical Activity Questionnaire – Short Form; SDQ: Strengths and Difficulties Questionnaire

### Family move app usage and child outcomes

The highest score accumulated over the 8-week intervention period was 9650 and the lowest score was 0. The average score was 1282 and the median (interquartile range) was 515 (1938). Among the 52 users, 9 received over the target score (i.e. 5000 points) and 8 received less than 100 points at the end of the intervention. Overall, 13 (25%) received more than 2060 points. Tables 5 and 6 presents the regression results between engagement with the Family Move app (indicated by participation ranking) and children’s IPAQ-SF score, CHQ-PF50 scale and summary scores, and SDQ subscale and total difficulties score among the 67 pairs at 1-month and 6-month follow-up. After adjusting for child gender, age and baseline value and recruitment month, higher participation ranking was significantly associated with lower SDQ Total Difficulties score at 1-month follow-up (β = − 0.15, *p* = 0.030).

**Table 5:** Association between Family Move app usage and child outcomes at one month (*n* = 67)

	B(95%CI)^a^	*p*-value	*R*^*2*^	F	*p*-value
**IPAQ-SF**					
Total physical activity level (MET-minutes/week)	−2.83(−46.49, 40.83)	0.897	0.55	10.94	< 0.001
Time spent in vigorous and moderate activities (hours/week)	1.17(−4.55, 6.89)	0.684	0.40	6.77	< 0.001
**CHQ-PF50**					
Physical functioning	0.01 (−0.09, 0.10)	0.924	0.53	14.06	< 0.001
Role functioning - emotional/behavior	−0.11 (− 0.37, 0.15)	0.413	0.60	18.03	< 0.001
Role functioning - physical	−0.02 (− 0.25, 0.21)	0.846	0.27	4.55	0.001
Bodily pain	0.04 (− 0.20, 0.28)	0.768	0.42	8.82	< 0.001
General behavior	0.12 (−0.11, 0.34)	0.304	0.62	19.56	< 0.001
Mental health	−0.07 (− 0.25, 0.11)	0.438	0.71	30.43	< 0.001
Self-esteem	0.10 (−0.07, 0.27)	0.256	0.62	20.10	< 0.001
General health perceptions	−0.06(− 0.29, 0.16)	0.575	0.51	12.54	< 0.001
Parental impact: emotional	−0.04(− 0.28, 0.20)	0.714	0.64	22.07	< 0.001
Parental impact: time	−0.04(− 0.29, 0.21)	0.746	0.71	30.18	< 0.001
Family activities	0.001(−0.30, 0.30)	0.996	0.54	14.27	< 0.001
Family cohesion	0.23(−0.10, 0.56)	0.165	0.54	14.05	< 0.001
Physical summary score	−0.03(− 0.12, 0.05)	0.465	0.55	15.07	< 0.001
Psychosocial summary score	−0.04(− 0.14, 0.07)	0.479	0.76	37.88	< 0.001
**SDQ**					
Emotional symptoms	−0.02(− 0.03, 0.004)	0.122	0.75	36.73	< 0.001
Conduct problems	−0.01(− 0.04, 0.009)	0.249	0.57	16.22	< 0.001
Hyperactivity/inattention	−0.02(− 0.04, 0.01)	0.169	0.72	31.89	< 0.001
Peer relationship problems	−0.02(− 0.04, 0.01)	0.138	0.52	15.22	< 0.001
Prosocial behavior	0.003(−0.02, 0.03)	0.816	0.52	15.12	< 0.001
Total difficulties	−0.06(− 0.11, − 0.01)	0.026	0.77	40.26	< 0.001

*CHQ-PF50* Child Health Questionnaire-Parent Form-50; *IPAQ-SF* International Physical Activity Questionnaire – Short Form; *SDQ* Strengths and Difficulties Questionnaire

^a^ Adjusted for child gender, age and baseline level and recruitment month

**Table 6:** Association between Family Move app usage and child outcomes at six months (*n* = 67)

	B(95%CI)^a^	*p*-value	*R*^*2*^	F	*p*-value
**IPAQ-SF**					
Total physical activity level (MET-minutes/week)	−0.29(−55.85, 55.27)	0.992	0.44	6.99	< 0.001
Time spent in vigorous and moderate activities (hours/week)	−0.61(−6.68, 5.47)	0.841	0.40	6.67	< 0.001
**CHQ-PF50**					
Physical functioning	0.06(−0.15, 0.26)	0.562	0.12	1.68	0.154
Role functioning - emotional/behavior	−0.06(− 0.36, 0.25)	0.717	0.50	11.94	< 0.001
Role functioning - physical	0.04(−0.21, 0.29)	0.739	0.31	5.48	< 0.001
Bodily pain	0.07(−0.17, 0.31)	0.581	0.31	5.57	< 0.001
General behavior	0.14(−0.11, 0.38)	0.259	0.50	12.09	< 0.001
Mental health	0.03(−0.18, 0.24)	0.754	0.61	18.67	< 0.001
Self-esteem	0.11(−0.05, 0.27)	0.185	0.71	29.12	< 0.001
General health perceptions	−0.01(− 0.22, 0.21)	0.942	0.55	15.13	< 0.001
Parental impact: emotional	0.17(−0.10, 0.43)	0.218	0.56	15.45	< 0.001
Parental impact: time	0.14(−0.18, 0.46)	0.374	0.55	14.66	< 0.001
Family activities	0.15(−0.18, 0.48)	0.379	0.43	9.03	< 0.001
Family cohesion	0.25(−0.14, 0.63)	0.209	0.40	7.85	< 0.001
Physical summary score	0.02(−0.10, 0.13)	0.785	0.39	7.79	< 0.001
Psychosocial summary score	0.02(−0.11, 0.14)	0.829	0.68	26.21	< 0.001
**SDQ**					
Emotional symptoms	−0.01(−0.03, 0.01)	0.585	0.73	33.68	< 0.001
Conduct problems	−0.02(− 0.04, 0.01)	0.145	0.40	8.17	< 0.001
Hyperactivity/inattention	−0.01(− 0.04, 0.01)	0.247	0.71	30.09	< 0.001
Peer relationship problems	−0.02(− 0.05, 0.01)	0.165	0.41	8.31	< 0.001
Prosocial behavior	−0.002(− 0.03, 0.03)	0.905	0.44	9.56	< 0.001
Total difficulties	−0.05(− 0.11, 0.01)	0.112	0.66	23.73	< 0.001

CHQ-PF50: Child Health Questionnaire-Parent Form-50; IPAQ-SF: International Physical Activity Questionnaire – Short Form; SDQ: Strengths and Difficulties Questionnaire

^a^ Adjusted for child gender, age and baseline level and recruitment month

## Discussion

This study examined changes in HRQOL, psychosocial wellbeing, and PA levels of children after the Family Move app-based interventions. We observed significant increases in the children’s total PA level and time spent in moderate and vigorous activities at 1-month follow-up. Significant improvement in psychosocial wellbeing and total PA level was observed at 6-month follow-up. Higher engagement with the Family Move app was significantly associated with fewer psychosocial problems at 1 month after the intervention. The findings suggest that the Family Move app-based intervention has the potential to improve children’s activity level and psychosocial outcomes through promoting parent-child exercises.

To the best of our knowledge, our study is the first to evaluate the effects of a mobile app intervention incorporating parent-child exercises with gaming elements on children. Other studies have examined mobile- or internet-based interventions to promote PA in adults and adolescents [28, 36, 37], but evidence for children was limited [38]. In a randomized controlled trial, young, male adolescents low in life satisfaction and self-rated health at baseline were found to benefit more from the gamified, mobile PA intervention [36]. Another 6-month internet-based PA intervention guided by the SCT successfully increased African American college students’ PA levels at 3 months after intervention, but these positive effects subsided at 6-month follow-up [28]. Consistent with these findings, our findings also show support for the use of technology to promote PA and improve health in children. It sheds light on the positive impact of partner exercise such as parent-child exercise in sustaining an exercise routine which has been underexplored in the literature.

The Family Move app intervention used a step-by-step approach to engage children and parents in the increasingly difficult exercise moves. At 1-month follow-up, we found significant improvement in PA levels, but the other outcomes showed minimal improvement. At 6-month follow-up, PA levels increased to a greater extent, and psychosocial wellbeing and perceived health also showed greater improvement. Previous research showed that participants were less likely to adhere to app use recommendations after the completion of the intervention [22, 39]. Some evidence also suggested that participants may regress to the pre-intervention value at a later follow-up time point [28]. However, we did not observe this trend, possibly because according to the SCT, starting with something that is easy and simple is a better strategy to help one achieve behavioral changes [40]. The aim of our intervention was to motivate children to become physically active by starting with simple exercise moves. Our results revealed an immediate and gradual increase in PA levels after the Family Move app-based intervention. This gradual improvement may account for better perceived health and fewer psychosocial problems at 6-month follow-up.

In addition, the gradual improvement in psychosocial wellbeing and perceived health might also be accounted by the low overall app usage. Our participants’ usage levels varied widely ranging from 0 to 9650 points, with only 25% obtaining more than 2060 points. The average change in psychosocial problems at 1 month after the intervention was not statistically significant, perhaps because most of the users had low to moderate app usage. On the other hand, higher app points were significantly associated with fewer psychosocial problems at 1-month follow-up. This could be because higher app points are equivalent to more video views which may indicate more frequent parent-child exercises. Previous studies have demonstrated an association between PA and psychosocial problems [41–43]. Consistent with these previous findings, the post-intervention increase in PA levels may account for greater reduction in psychosocial problems among children who used the app more frequently during the intervention period. On the other hand, the significant reduction in psychosocial problems at 6-month follow-up was not related to the app usage during the intervention period, perhaps because frequent and non-frequent users may establish a similar exercise routine leading to similar levels of psychosocial problems over time, although this speculation should be substantiated in future research.

### Strengths and limitations

The strength of this intervention study is its ability to measure the participant’s engagement with the app. We used the total number of points accumulated over the intervention period as an indicator of participants’ engagement with the app which has been rarely demonstrated in previous research [44, 45]. Future family PA interventions can be built upon the framework and materials used in this intervention. Despite the strengths, this study has several limitations. First, the sample was small and consisted of predominantly active and male children from higher income families, which reduced the generalizability of our study findings to other populations. Second, we used parent proxy-report measures to assess child PA level, HRQOL, and psychosocial wellbeing which could be subject to bias. Future research might consider the use of self-report measures to assess adolescent children’s outcomes and the use of wearable devices to obtain objective PA data. Third, we did not use any app-based monitoring strategies to ensure that parents and children watched the video or performed the exercises together. Finally, we lacked a control group to eliminate potential confounding effects which might have influenced the results. Furthermore, high attrition at 6-month follow-up may pose a threat to the validity of the findings. An adequately powered trial should be carried out to affirm the effectiveness of the Family Move app as a health promotion tool.

## Conclusion

This study provides preliminary evidence that a mobile app-based intervention integrating simple parent-child exercise moves with gaming elements has the potential to promote PA and reduce psychosocial problems in children. Engaging parent and children together in exercise through points and level system can bring joy and fun which may further increase the chance of intervention success. This study also demonstrates how to use points to quantify participants’ level of adherence to the intervention which is crucial for disentangling the intervention mechanism. Future research should use a trial design to ascertain the intervention effects and its theoretical framework perhaps by measuring the constructs such as self-efficacy. New strategies should also be developed to upgrade the app to increase registration and usage.

## Supplementary information

**Additional file 1.** Details of exercise demo clips.

**Additional file 2.** Screenshots and functions of the Family Move app.

**Additional file 3.** User app points and ranking.

## Data Availability

The datasets generated and/or analyzed during the current study are available from the corresponding author on request.

## Abbreviations

Apps: Applications
CHQ-PF50: Child health questionnaire – parent form – 50
CMS: Central management system
HRQOL: Health-related quality of life
IPAQ-SF: International physical activity questionnaire – short form – chinese version
ITT: Intention-to-treat
PA: Physical activity
SDQ: Strength and difficulties questionnaire
SCT: Social cognitive theory
