# Supplementary material for: Development and pilot evaluation of a mobile app on parent-child exercises to improve physical activity and psychosocial outcomes of Hong Kong Chinese children
Source: BMC Public Health. 2020 Oct 14;20:1544. doi: 10.1186/s12889-020-09655-9 (PMC7556926; doi:10.1186/s12889-020-09655-9)
Supplement: Supplementary file 1 — Additional file 1. Details of exercise demo clips. [file 12889_2020_9655_MOESM1_ESM.docx]

Additional file 1: Details of exercise demo clips

| **Exercises Demo Clips**  **(Age 6-10 years)** | **Exercises Demo Clips**  **(Age 11 years or above)** | **Time**  **(Seconds)** | **Assigned Point Value** |
| --- | --- | --- | --- |
| **Level 1** | | | |
| Warm-up (swimming, shrug, shoulder rotation, skipping rope, and running in place) | Warm-up (swimming, shrug, shoulder rotation, skipping rope, and running in place) | 60 | 20 |
| Forward and backward lunge | Forward and backward lunge | 30 | 10 |
| Partner jumping jack | Partner jumping jack | 30 | 10 |
| Back to back pass pillow | Plank hand slap | 30 | 10 |
| Alternate foot jump (holding hands) | Roller skate | 30 | 10 |
| Three-minute challenge (including all four individual exercises) | Three-minute challenge (including all four individual exercises) | 180 | 60 |
| **Level 2** | | | |
| Warm-up (deltoid stretch, triceps stretch, and alternate foot jump) | Warm-up (deltoid stretch, triceps stretch, and alternate foot jump) | 60 | 40 |
| Standing cross crunch + slap hands | Lateral jump + slap hands | 30 | 20 |
| Plank hand slap | Standing cross crunch + slap hands | 30 | 20 |
| Sit up & pass a pillow | High knee + butt kick | 30 | 20 |
| High knee + butt kick | Sit up + pass a pillow | 30 | 20 |
| Three-minute challenge (including all four individual exercises) | Three-minute challenge (including all four individual exercises) | 180 | 120 |
| **Level 3** | | | |
| Warm-up (side bend, quadriceps stretch, and lunge) | Warm-up (side bend, quadriceps stretch, and lunge) | 60 | 60 |
| Lateral jump + slap hands | Squat jump + high five | 30 | 30 |
| Jump (holding hands) | Push-up + high five | 30 | 30 |
| Skating and touching the ground | Squat + overhead pass | 30 | 30 |
| Squat jump + butt kick | Burpee | 30 | 30 |
| Three-minute challenge (including all four individual exercises) | Three-minute challenge (including all four individual exercises) | 180 | 180 |
