# Supplementary material for: Development and pilot evaluation of a mobile app on parent-child exercises to improve physical activity and psychosocial outcomes of Hong Kong Chinese children
Source: BMC Public Health. 2020 Oct 14;20:1544. doi: 10.1186/s12889-020-09655-9 (PMC7556926; doi:10.1186/s12889-020-09655-9)
Supplement: Supplementary file 2 — Additional file 2. Screenshots and functions of the Family Move app. [file 12889_2020_9655_MOESM2_ESM.docx]

Additional file 2: Screenshots and functions of the Family Move app

| **Screenshots** | **Description** |
| --- | --- |
| 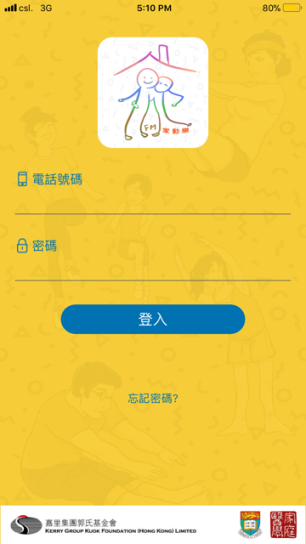 | ***A unique, anonymous participant number was assigned to each user family account****.* This allows the administrator to monitor and analyze user data such as frequency and duration of app use. |
| 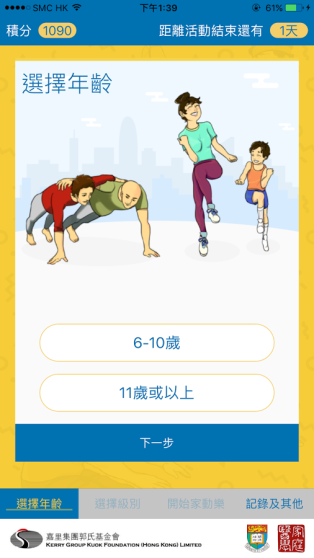  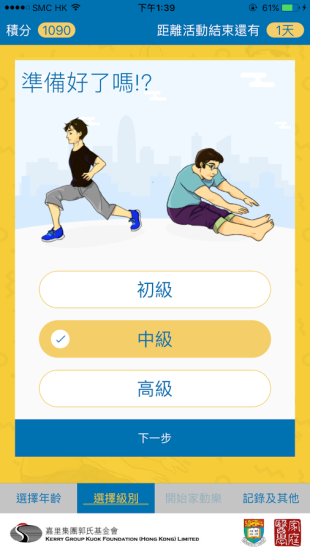 | ***An automated 8-week parent-child exercise program with age-appropriate exercises organized by levels of exercise difficulty****.* Exercise moves are stratified into two groups for children aged 6 to 10 years and adolescents aged 11 years or above, respectively. The exercise moves within each age group are further subdivided into three levels of difficulty. Each level has a different release period (i.e. release of clips in *Level 1* in Week 1, those in *Level 2* in Week 3, those in *Level 3* in Week 5, and revision of all clips in Week 7). |
| 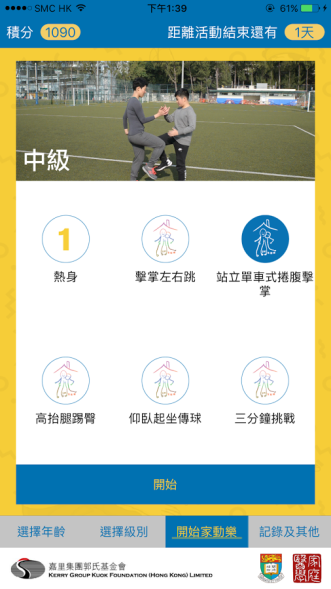 | ***A set of exercise demo videos presented by age group and levels***. Each level consists of one warm-up clip (1 minute), 4 short demo clips (30 seconds per clip) including Cantonese audio instructions and subtitles to remind users of the benefits and correct posture of the exercise move, and 1 long challenge clip (3 minutes) integrating the four moves with background music. |
| 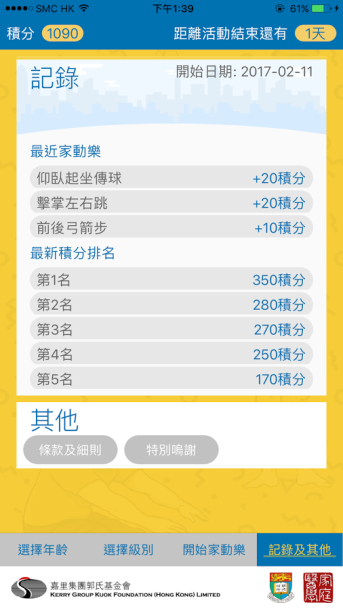 | ***Point system*.** Users receive points for each exercise clip viewed. More points are awarded to more difficult moves. Points can be accumulated and used to rank the level of app usage.   - **Scoreboard.** A scoreboard is used to display personal score and the scores of the top five users. This can facilitate self-regulatory processes by comparing usage levels among users. |
| ***Push notifications***. Notifications are pushed twice per week as prompts to remind users of the health benefits of practicing the exercise moves. | |
