# Supplementary material for: Development and pilot evaluation of a mobile app on parent-child exercises to improve physical activity and psychosocial outcomes of Hong Kong Chinese children
Source: BMC Public Health. 2020 Oct 14;20:1544. doi: 10.1186/s12889-020-09655-9 (PMC7556926; doi:10.1186/s12889-020-09655-9)
Supplement: Supplementary file 3 — Additional file 3. User app points and ranking. [file 12889_2020_9655_MOESM3_ESM.docx]

Additional file 3: User app points and ranking

| Rank | Score | Number of users | Rank | Score | Number of users |
| --- | --- | --- | --- | --- | --- |
| 0 | 0 | 15 | 22 | 580 | 1 |
| 1 | 10 | 1 | 23 | 730 | 1 |
| 2 | 20 | 2 | 24 | 820 | 1 |
| 3 | 40 | 2 | 25 | 990 | 1 |
| 4 | 50 | 1 | 26 | 1190 | 1 |
| 5 | 60 | 1 | 27 | 1390 | 1 |
| 6 | 90 | 1 | 28 | 1850 | 1 |
| 7 | 110 | 1 | 29 | 1870 | 1 |
| 8 | 120 | 4 | 30 | 1940 | 1 |
| 9 | 130 | 2 | 31 | 2100 | 1 |
| 10 | 140 | 2 | 32 | 2240 | 1 |
| 11 | 150 | 1 | 33 | 2850 | 1 |
| 12 | 160 | 1 | 34 | 3780 | 1 |
| 13 | 200 | 1 | 35 | 5020 | 1 |
| 14 | 220 | 2 | 36 | 5070 | 1 |
| 15 | 280 | 1 | 37 | 5130 | 1 |
| 16 | 300 | 1 | 38 | 5260 | 1 |
| 17 | 390 | 1 | 39 | 5990 | 2 |
| 18 | 510 | 1 | 40 | 6220 | 1 |
| 19 | 520 | 1 | 41 | 9220 | 1 |
| 20 | 550 | 1 | 42 | 9650 | 1 |
| 21 | 570 | 2 |  |  |  |
